# Preparing for Telemedicine Visits: Guidelines and Setup

**DOI:** 10.3389/fmed.2020.600794

**Published:** 2020-11-25

**Authors:** Aman Prasad, Ryan Brewster, Divya Rajasekaran, Karthik Rajasekaran

**Affiliations:** ^1^Perelman School of Medicine, University of Pennsylvania, Philadelphia, PA, United States; ^2^Stanford University School of Medicine, Stanford University, Stanford, CA, United States; ^3^Department of Endocrinology, Summit Medical Group, Berkeley Heights, NJ, United States; ^4^Department of Otorhinolaryngology – Head and Neck Surgery, Perelman School of Medicine, University of Pennsylvania, Philadelphia, PA, United States

**Keywords:** telemedicine, virtual medicine, COVID-19, practice guidelines, patient handout

## Introduction

In response to the 2019 coronavirus disease (COVID-19), many medical practices have canceled outpatient visits in an effort to protect healthcare workers and the public. As an efficient way to maintain chronic care for patients, physicians have turned to telemedicine visits for outpatient appointments as a way to maximize their social distancing (1–3). Physicians can use a variety of telecommunication services, such as Zoom (San Jose, CA), Doximity (San Francisco, CA), etc., to virtually meet with patients and perform basic assessments for future follow-up. Telemedicine has become a mainstay of patient care in the medical climate of the COVID-19 pandemic. Given its many benefits, such as convenience, time efficiency, cost savings, and ease of use, telehealth services may continue to grow in popularity for some time (4, 5). It is worth noting, however, that there are apparent limitations to the use of remote visits, namely the inability to conduct a comprehensive physical exam and to perceive non-verbal communication. We do not assert that telemedicine represents a new paradigm of all future patient care. However, given the recent resurgence of COVID-19 cases in many states, telehealth services may play a major role in healthcare for longer than initially expected.

With rapid implementation of telemedicine, there has been very little time for physicians and patients to fully adjust to this new medium of patient care. In order to maximize the efficiency, utility and value of these telemedicine visits, it is crucial for both parties to be properly educated and prepared. Previous studies have shown high patient satisfaction with ambulatory telemedicine visits, however patients frequently noted overall setup, technological barriers, and appointment expectations as points for future improvement (6–9). Thus, quality improvement initiatives aimed at increasing patient and physician preparedness for virtual medical appointments will play a large role in maximizing patient satisfaction for such visits, which has been shown to impact the overall success and adoption of telemedicine programs (10). Our group has previously designed and distributed a set of guidelines and a patient-facing handout in order to facilitate more effective, time efficient telemedicine visits for otolaryngology patients (11). In the authors' experience, use of this handout has vastly improved patient preparedness for such appointments. Its use has since been expanded to the entire department and patient satisfaction with telemedicine visits has also vastly improved (6, 12). To our knowledge, such a handout or graphic has not been developed or distributed in other medical fields. In this paper, we aim to provide the medical community with a useful patient handout on telemedicine preparedness that can be applied to a variety of specialties. In addition, we further outline and elaborate on such expectations on the side of both healthcare providers and patients.

## Guidelines for Healthcare Providers

**Figure 1:**
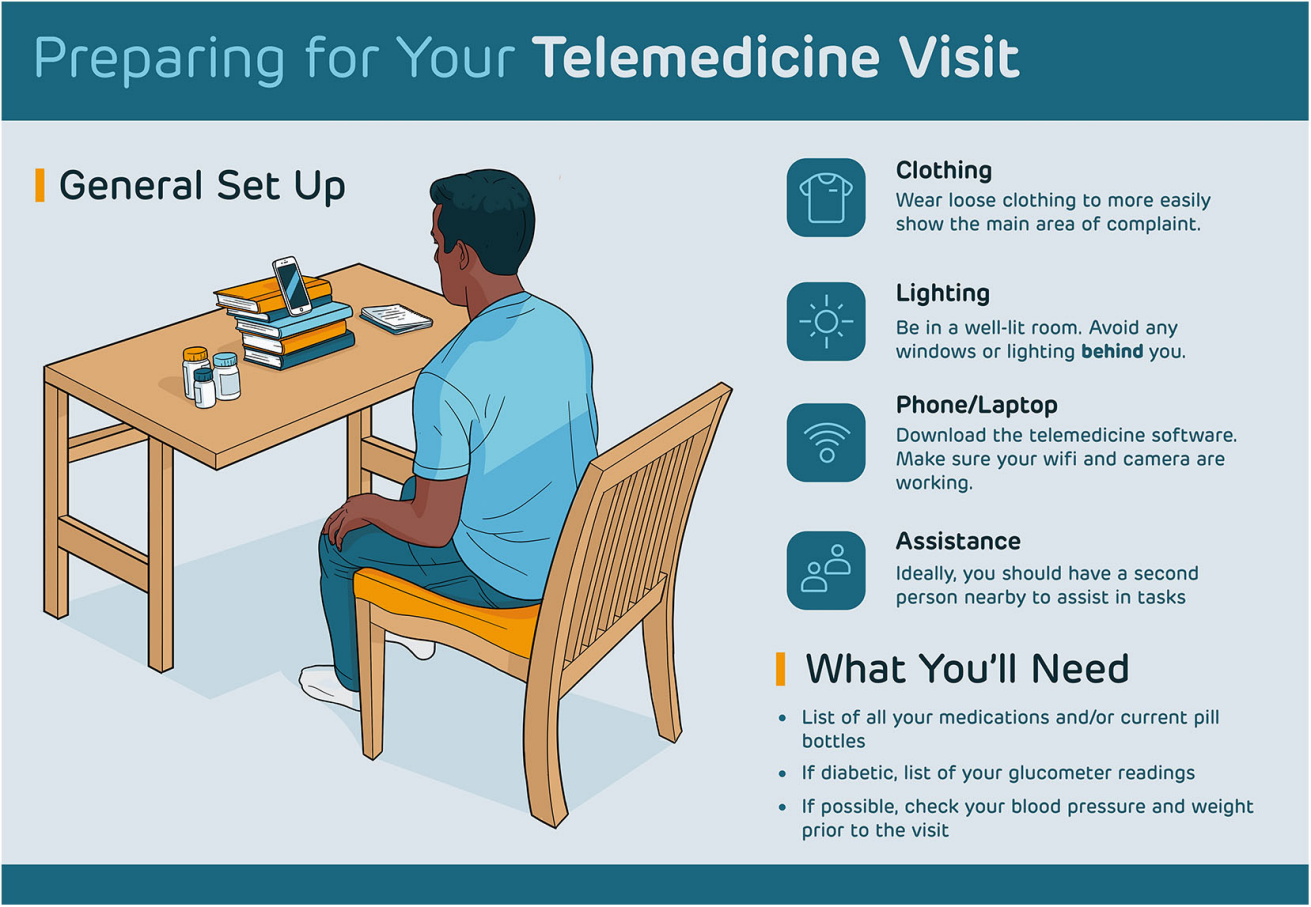
Telemedicine handout.

Firstly, physicians should ensure their technological setup is secure and professional prior to any appointment. This includes maintaining a robust internet connection, having clear video, and smooth audio. While verbal aspects of obtaining patient history is largely unimpaired through a telemedicine visit, aspects of the physical exam are much more difficult to accomplish. If the physician asks a patient to perform aspects of a physical exam, the physician should first attempt to demonstrate such maneuvers on themselves first. This will mitigate any lapses in communication between physicians and patients during such tasks. Additionally, physicians' administrative staff should contact the patient at least a day in advance of their appointment in order to provide them with adequate setup information. This could include distributing a clear, concise handout such as the one provided in Figure 1. Such communication provides patients ample opportunity to gather materials and become familiarized with specific expectations for the visit. Lastly, physicians may find it appropriate to add other consult services, such as social workers or nurse navigators, to the video call for patients dealing with complex issues. In this way, telemedicine visits can act as a multidisciplinary tool for patients to interact with providers in a single, unified setting.

## Guidelines for Patients

### Positioning

Similar to guidelines published previously (11), we advise patients to sit on a stable chair with good posture. Patients should also sit in a room with proper lighting, avoiding light sources positioned behind their backs as this can lead to a silhouette effect. These preparations will ensure that physicians have a clear view of patients throughout the visit, which can be difficult to maintain in cramped or dim conditions. The patients' video camera should also be placed as close to eye-level as possible. If patients utilize phones or iPads for their visit, they may be instructed to support these devices on books or other materials around the house in order to keep both hands free for possible physical exam tasks physicians may request patients to perform (see Figure 1).

### Equipment

Patients should be notified of any specific equipment they may need prior to the telemedicine visit. A laptop, phone, or tablet would be necessary for conduction of the telemedicine visit itself. Patients should ensure these devices work properly prior to the visit as well. This can be accomplished by video-chatting a family member or friend to ensure the microphone and camera are functioning properly. If accessible, having a blood pressure cuff, glucometer, or weighing scale may be helpful in order to record rudimentary vital signs. Lastly, patients should be prepared with an updated and comprehensive list of active medications to aid the physician during the history taking process (see Figure 1).

### Additional Considerations

If requested by the physician, patients should be prepared with recent logs of blood pressure readings, blood glucose levels (from glucometer readings), and weight. If possible, patients should have a family member or friend present during the visit to assist with aspects of the physical exam or with camera positioning onto an area of interest. If patients have scheduled the visit to examine a certain body part of interest, they should take care to wear loose clothing that can allow that area to be easily visible if needed. A supplementary light source such as a flashlight can aid in such visualization over video as well. In this case, physicians can ask a family member or friend of the patient, if present, to assist in focusing the light source if the patient finds it difficult (Figure 1).

## Conclusion

Due to social distancing policies enacted as a result of COVID-19, the prevalence of virtual ambulatory visits has rapidly grown. Such visits may remain popular among healthcare providers with the recent resurgence of COVID-19 cases in the United States. Using the authors' experience and previous literature, we have provided a basic framework with which all medical practices can prepare for their telemedicine visits. In particular, we have designed a graphical guide for patients meant to be distributed by practices to improve patient awareness and increase appointment efficiency. By providing this structure, it formalizes the telemedicine protocols to more accurately represent an in-person physician appointment. Given the recent, widespread adoption of this practice, these guidelines can be used as a basis for telemedicine visits even beyond the COVID-19 pandemic.

## Author Contributions

All authors provided substantial contributions to the conception of work, critical revisions for important intellectual content, final approval of version, and agreement to be accountable for all aspects of the work.

## Conflict of Interest

The authors declare that the research was conducted in the absence of any commercial or financial relationships that could be construed as a potential conflict of interest.
